# Efficacy and radiographic analysis of oblique lumbar interbody fusion in treating adult spinal deformity

**DOI:** 10.1371/journal.pone.0257316

**Published:** 2021-09-10

**Authors:** Dae-Jean Jo, Eun-Min Seo

**Affiliations:** 1 Department of Neurosurgery, Spine Center, Kyung Hee University Hospital at Gangdong, Seoul, Korea; 2 Department of Orthopedic Surgery, Chuncheon Sacred Heart Hospital, Hallym University College of Medicine, Chuncheon, Korea; University of California San Francisco, UNITED STATES

## Abstract

Adult spinal deformity (ASD) is usually rigid and requires a combined anterior–posterior approach for deformity correction. Anterior lumbar interbody fusion (ALIF) allows direct access to the disc space and placement of a large interbody graft. A larger interbody graft facilitates correction of ASD. However, an anterior approach carries significant risks. Lateral lumbar interbody fusion (LLIF) through a minimally invasive approach has recently been used for ASD. The present study was performed to evaluate the effectiveness of oblique lumbar interbody fusion (OLIF) in the treatment of ASD. We performed a retrospective study utilizing the data of 74 patients with ASD. The inclusion criteria were lumbar coronal Cobb angle > 20°, pelvic incidence (PI)–lumbar lordosis (LL) mismatch > 10°, and minimum follow–up of 2 years. Patients were divided into two groups: ALIF combined with posterior spinal fixation (ALIF+PSF) (*n* = 38) and OLIF combined with posterior spinal fixation (OLIF+PSF) (*n* = 36). The perioperative spinal deformity radiographic parameters, complications, and health-related quality of life (HRQoL) outcomes were assessed and compared between the two groups. The preoperative sagittal vertical axis (SVA), LL, PI–LL mismatch, and lumbar Cobb angles were similar between the two groups. Patients in the OLIF+PSF group had a slightly higher mean number of interbody fusion levels than those in the ALIF+PSF group. At the final follow–up, all radiographic parameters and HRQoL scores were similar between the two groups. However, the rates of perioperative complications were higher in the ALIF+PSF than OLIF+PSF group. The ALIF+PSF and OLIF+PSF groups showed similar radiographic and HRQoL outcomes. These observations suggest that OLIF is a safe and reliable surgical treatment option for ASD.

## Introduction

Loss of sagittal and coronal balance is associated with pain and disability. Its correction is the goal of surgical treatment for patients with adult spinal deformity (ASD) [1–3]. Surgical options for ASD vary according to the type of deformity. Patients with mild deformity may benefit from a posterior–only approach [4]. However, moderate–to–severe ASD generally requires both anterior and posterior approaches [3–5]. The anterior approach allows direct access to the disc space and placement of a large interbody graft, where a larger interbody graft facilitates correction of ASD [3, 6]. However, the anterior approach carries significant risks (retrograde ejaculation, great vessel injury, ureteral trauma, and prolonged ileus) [7–12]. Lateral lumbar interbody fusion (LLIF) through a minimally invasive approach has recently been used for ASD [13–18].

LLIF is divided into direct lateral interbody fusion (DLIF) and oblique lumbar interbody fusion (OLIF). DLIF is performed by a lateral retroperitoneal transpsoas approach. DLIF can damage the neural structures and psoas muscle, and can also cause lower limb weakness or paresthesia [19]. OLIF has several potential advantages, including reduced invasion of the psoas muscle and neural structures, and relatively good access to lower lumbar levels. However, access to the lower lumbar levels is restricted in some cases involving a high–riding pelvis [20–22]. Consequently, to perform lumbar interbody fusion from L1 to S1, a separate incision and/or position change are generally required [23]. We have performed lumbar interbody fusion from L1 to S1 without a separate incision or position change by tilting the operating table in a 45° right oblique decubitus position [24].

The present study was performed to evaluate the effectiveness of OLIF in the treatment of ASD. Radiographic data, complications, and health–related quality of life (HRQoL) outcomes of patients with ASD undergoing anterior lumbar interbody fusion (ALIF) combined with posterior spinal fixation (ALIF+PSF) were then assessed and compared to those of patients undergoing OLIF combined with posterior spinal fixation (OLIF+PSF).

## Materials and methods

This study was approved by the Investigational Review Board (IRB) for the Protection of Human Subjects of Chuncheon Sacred Heart Hospital, Hallym University College of Medicine.

All subjects provided informed consent prior to participation in accordance with procedures approved by the IRB.

### Patients

Medical records of consecutive adults (aged > 45 years) who underwent surgery for ASD at a single institution between 2009 and 2019 were reviewed retrospectively. A total of 196 ASD patients underwent operations performed by the same surgeon. Indications for surgeries included symptomatic back and/or leg pain attributed to ASD that was unresponsive to conservative treatment. Patients undergoing pedicle subtraction osteotomy (PSO) or posterior vertebral column resection (PVCR), and those with a spinal deformity related to infection, incomplete data, or who did not undergo lumbar interbody fusion were excluded. The inclusion criteria were lumbar coronal Cobb angle > 20°, treatment of two or more disc levels, pelvic incidence (PI)–lumbar lordosis (LL) mismatch > 10°, and minimum follow–up of > 2 years. A total of 74 patients were included in the analysis. The patients were divided into two groups according to surgical technique: ALIF+PSF (*n* = 38) and OLIF+PSF (*n* = 36).

### Operative technique

To minimize the influence of surgical technique on outcomes, one senior surgeon performed all operations. Unlike adolescent spinal deformities, adult spinal deformities are usually rigid and require a combined anterior-posterior approach for deformity correction [24].

**Fig 1:**
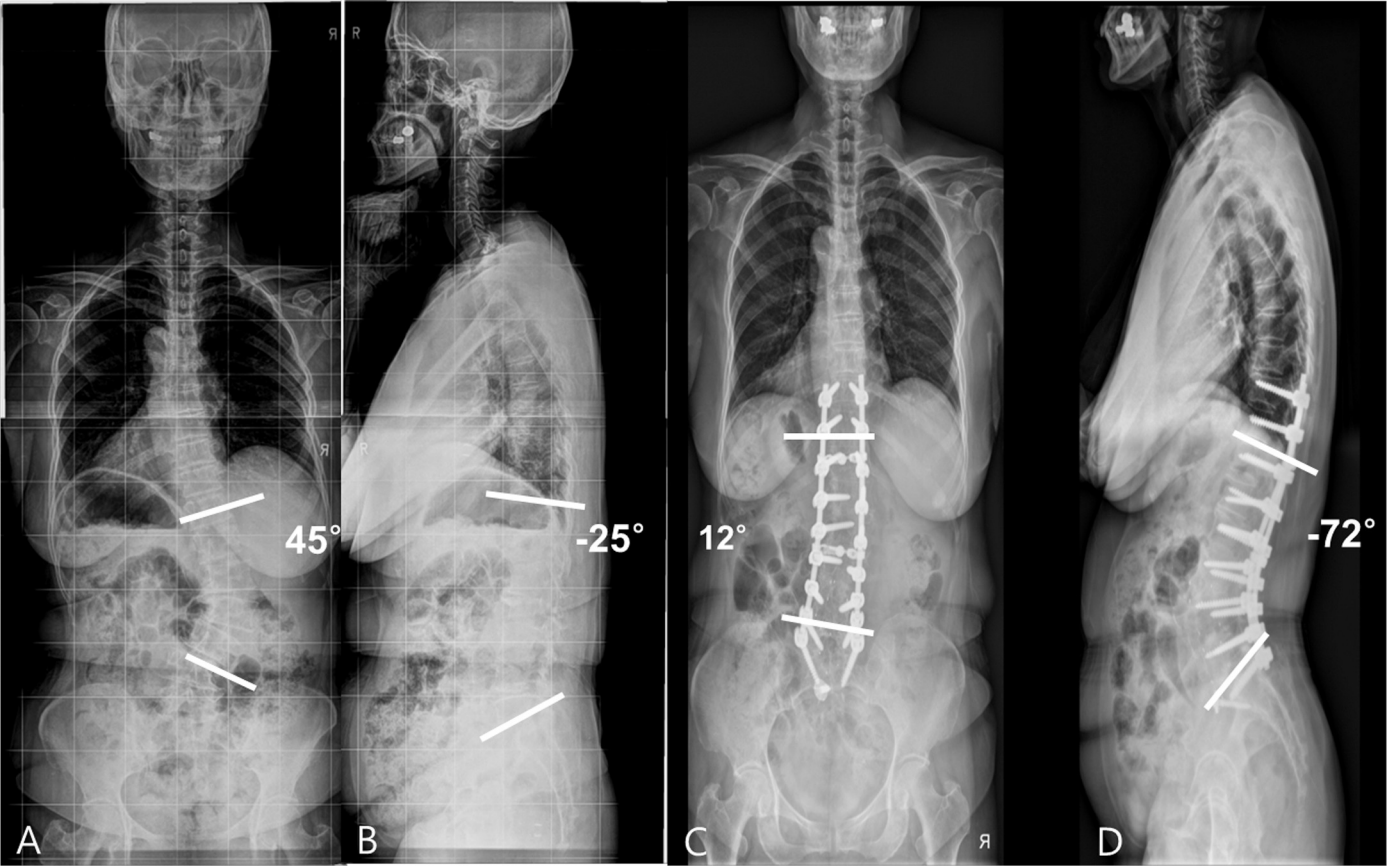
(A, B) Anteroposterior (AP) and lateral standing radiographs of a 65–year–old woman with severe back pain and pain in both legs, showing degenerative lumbar kyphoscoliosis. A three–step (posterior–anterior–posterior) approach was used to obtain adequate sagittal and coronal balance. (C, D) Postoperative AP and lateral radiographs showed L3–S1 interbody fusion and good sagittal alignment.

Two techniques were used throughout the study period. OLIF was introduced after ALIF. We planned a three–step (posterior–oblique or anterior–posterior) approach to achieve adequate sagittal and coronal balance. In the first step, pedicle screw insertion and posterior release (facetectomy and/or laminectomy) were performed. The autologous bone obtained from the laminar, articular facet, and spinous process was stored in the bone bank and used when interbody fusion was performed. In the second step, multi–level ALIF or OLIF was performed. For ALIF, the patient was placed in the supine position. After fluoroscopic identification of the operation level, a midline skin incision was made. A retroperitoneal approach to the appropriate disc space was achieved. After careful dissection and retraction of abdominal vessels, the anterior longitudinal ligament, disc material, and posterior annulus were removed. A polyetheretherketone cage filled with auto–allograft was inserted (Fig 1).

**Fig 2:**
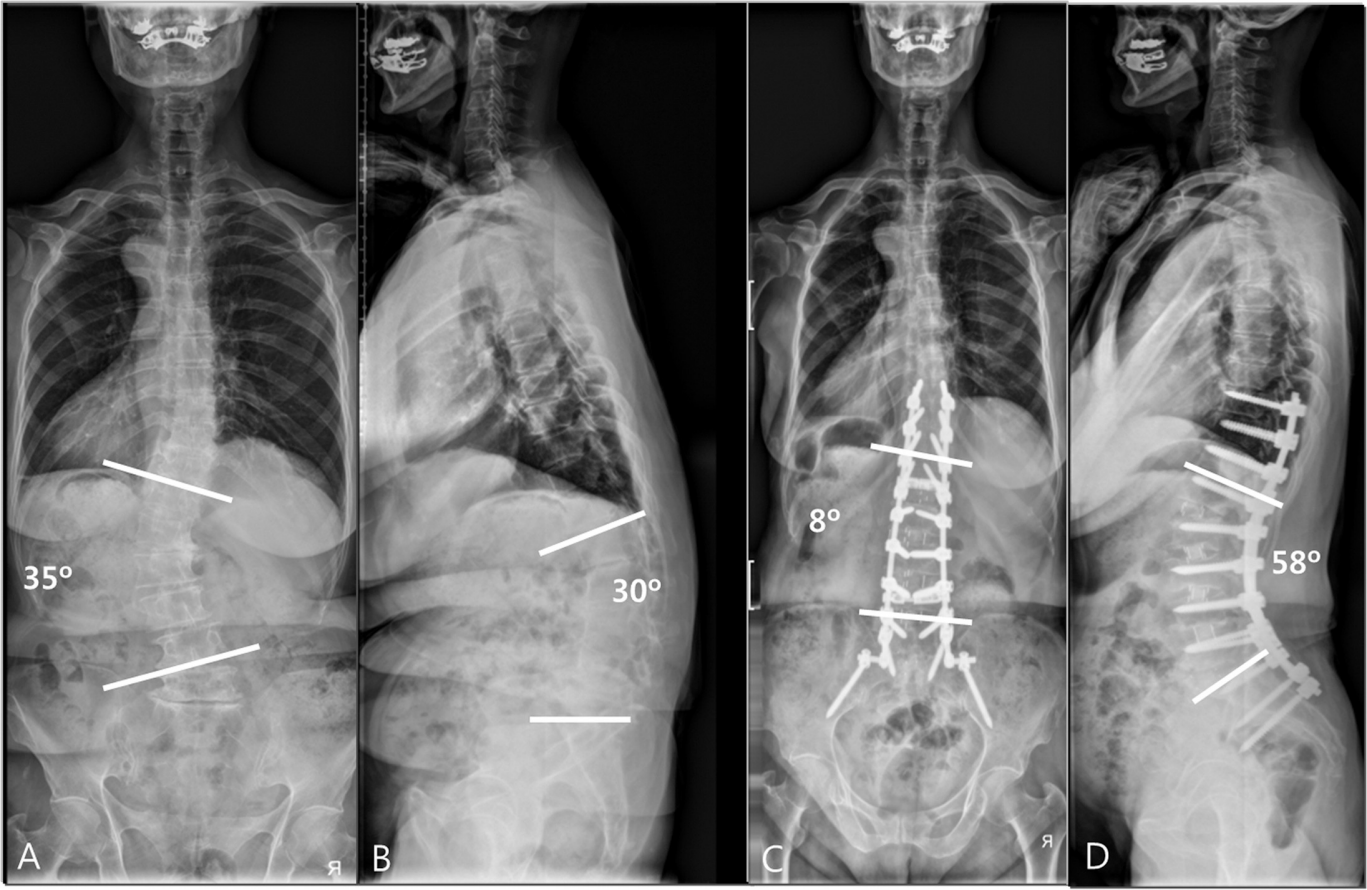
(A, B) Anteroposterior (AP) and lateral standing radiographs of a 70–year–old woman who had severe back pain and pain in both legs, showing degenerative lumbar kyphoscoliosis. A three–step (posterior–oblique–posterior) approach was used to obtain adequate sagittal and coronal balance. (C, D) Postoperative AP and lateral radiographs showed L1–5 interbody fusion and good sagittal alignment.

For OLIF, the patient was placed in a 45° right oblique decubitus position for left–side elevation When L1–L5 interbody fusion was performed, the operating table was maximally tilted to the contralateral side, such that the patient’s position was similar to the direct lateral position [24]. When L5–S1 interbody fusion was performed, the operating table was tilted to place the patient in the supine position [23]. The retroperitoneum was approached by sweeping the posterior peritoneum away from the psoas muscle and iliac vessels. The great vessels were retracted anteromedially to expose the intervertebral disc space. A dissection plane was developed between the left lateral border of the aorta (or iliac artery) and anterior border of the psoas muscle, which was retracted posteriorly. After each disc space had been exposed, a disc level check was performed with a guide pin and C–arm. Discectomy was performed posterior to the anterior longitudinal ligament. A trapezoid–shaped polyetheretherketone cage (Clydesdale; Medtronic Inc., Minneapolis, MN) with auto–allograft was then inserted in a slightly oblique manner [26]. During the last step, posterior rod assembly and posterior fusion were performed. LL was restored using a rod cantilever and compression technique (Fig 2).

A long operative time due to this three–step approach could increase early perioperative complications. Therefore, the three–step (posterior–anterior or oblique–posterior) approach was performed at two different time points within 1 week. The second step and third steps were performed 1 week later.

### Radiologic evaluation and clinical assessments

Demographic and clinical data, including patient age, sex, bone mineral density (BMD), comorbidities, number of interbody fusion levels, incidence of implant failure (proximal vertebra fracture, screw pullout, rod fracture), pseudarthrosis, and perioperative complications, were recorded. Radiological parameters were measured on 36–inch standing radiographs obtained preoperatively, immediate postoperatively, and at the most recent follow–up. Coronal and sagittal alignment was assessed, including the C7–S1 sagittal vertical axis (SVA; defined as the distance from the posterosuperior corner of the S1 body to the C7 plumb line), thoracic kyphosis (TK; defined as the sagittal Cobb angle from the superior endplate of T–5 to the inferior endplate of T–12), LL (defined as the angle formed by two parallel lines; one parallel to the superior endplate of L1 and the other to the superior endplate of S1), PI (defined as the angle subtended by a line drawn perpendicular to the superior endplate of S1 and a line drawn from the center of the femoral head to the midpoint of the superior endplate of S1), PT (defined as the angle made between lines originating at the bicoxofemoral axis and extending vertically to the middle of the superior endplate of S–1), PI–LL mismatch, and the coronal Cobb angle of the lumbar curves.

Fusion status was determined in plain neutral and flexion-extension radiographs, and in computed tomography scans in cases for which radiographs were inconclusive. Fusion was defined as bridging bone connecting adjacent vertebral bodies, either through or around implants with angular motion < 5°and translation ≤ 3 mm.

Surgical time, operative blood loss, and perioperative complications were assessed. For clinical outcome assessment, HRQoL was analyzed using a visual analog scale (VAS) for back and leg pain, the Oswestry Disability Index (ODI), and the Scoliosis Research Society–22 (SRS–22) questionnaire. The SRS–22 provides a total score based on five subdomains: pain, function, self–image, mental health, and satisfaction [25].

### Statistical analysis

Measurements were performed by two independent observers using a PACS system (π view®; Infinitt, Seoul, Korea). Intraobserver and interobserver agreement rates between the two observers were evaluated by calculating κ values. For statistical analysis, SPSS software (ver. 22.0; SPSS Inc., Chicago, IL, USA) was used. In all analyses, *P* < 0.05 was taken to indicate statistical significance. Continuous variables are presented as means ± SD. Frequency analysis was used for categorical variables. ANOVA and the Kruskal–Wallis test were used as appropriate for group comparisons.

## Results

The interobserver agreement rate was 94% (mean κ = 0.75), and the intraobserver agreement rate was 97% (mean κ = 0.81); thus, there was good agreement.

A total of 74 patients met the inclusion criteria. The cohort included 65 women and 9 men with a mean age of 69 ± 8 years and mean follow–up period of 33.3 ± 21.1 months. There were no differences in mean age or follow–up period between the ALIF+PSF group (*n* = 38) and OLIF+PSF group (*n* = 36). The preoperative SVA, LL, PI–LL mismatch, lumbar Cobb angle, and thoracic Cobb angle were similar between the two groups. The OLIF group had a slightly higher mean interbody fusion level (3.25 ± 1.2 levels) than the ALIF group (2.92 ± 0.8 levels). There were 3 cases at the L1–5 level, 3 at the L1–S1 level, 4 at the L2–4 level, 11 at the L2–5 level, 2 at the L2–S1 level, 8 at the L3–5 level, 5 at the L3–S1 level, and 2 at the L4–S1 level in the ALIF group, and 7 at the L1–5 level, 3 at the L1–S1 level, 2 at the L2–4 level, 13 at L2–5 level, 3 at the L2–S1 level, 4 at the L3–5 level, 3 at the L3–S1 level, and 1 at the L4–S1 level in the OLIF group. The mean posterior fusion level was not significantly different between the ALIF and OLIF groups (5.2 ± 3.6 vs. 5.7 ± 3.4). There were 4 cases at the T10–S1 level, 2 at the T10–ilium level, 16 at the L1–S1 level, 5 at the L1–ilium level, 4 at the L1–5 level, and 7 at the L2–S1 level in the ALIF group, and 5 at the T10–S1 level, 5 at the T10–ilium level, 5 at the L1–S1 level, 17 at the L1–ilium level, 3 at the L1–5 level, and 4 at the L2–S1 level in the OLIF group. Demographic data are presented in Table 1.

**Table 1:** Summary of demographic profile.

Dermographics	All patients	ALIF	OLIF	P—value
No. of patients	74	38	36	0.755
Age(years)	69 ± 8	67.4±7.6	69.7±6.9	0.063
Gender(M:F)	9:65	4:34	5:31	0.323
BMD T-score	− 2.4 ± 0.5	− 2.5 ± 0.3	− 2.4 ± 0.5	0.073
Follow-up (months) (mean ± SD)	33.3 ± 21.1	34.9±22.6	27.7± 21.7	0.062
Interbody fusion levels (mean ± SD)	3.02±1	2.92 ± 0.8	3.25 ± 1.2	0.026
Posterior fusion levels (mean ± SD)	5.4 ± 3.4	5.2 ± 3.6	5.7 ± 3.4	0.075
SVA (%)				0.622
≤10 cm	32	28.6	35.2	
> 10 cm	68	71.4	64.8	
PI-LL mismatch (%)				0.634
>10° but ≤20°	37.6	38.5	36.7	
>20°	62.4	61.5	63.3	
PT (%)				0.652
≤20°	27.2	23.1	31.3	
>20° but <30°	45	46.2	43.8	
≥30°	27.8	30.7	24.9	

### Radiographic outcomes

The outcome measures are summarized in Table 2. The preoperative SVA, LL, PI–LL mismatch, thoracic kyphosis, and lumbar Cobb angles of both groups were similar. The postoperative and final follow–up SVA, LL, PI–LL mismatch, and lumbar Cobb angles were similarly improved in both groups. At the final follow–up, although the SVA, LL, PI–LL mismatch, and lumbar Cobb angles decreased slightly in both groups, these decreases were not statistically significant.

**Table 2:** Comparisons of sagittal and coronal radiographic data between the two groups.

	ALIF	OLIF	P—value
Thoracic kyphosis (°)			
Preoperation	24.2±16.8	23.3±12.3	0.182
Postoperation	32.5±12.9	25.6±12.9	0.248
Final follow up	36.2±15.0	28.0±10.8	0.449
p value (pre-final)	<0.001	<0.001	
Lumbar lordosis(°)			
Preoperation	30.5±15.3	28.3±23.9	0.865
Postoperation	49.5±13.9	50.2±19.5	0.208
Final follow up	48.5±13.4	48.7±20.9	0.484
p value (pre-final)	<0.001	<0.001	
PI (°)			
Preoperation	52.1±12.6	51.7±6.6	0.863
Postoperation	53.8±12.9	52.2±5.9	0.759
Final follow up	53.6±10.6	52.4±5.6	0.768
p value (pre-final)	0.148	0.672	
PT (°)			
Preoperation	25.6±11.4	24.8±9.6	0.339
Postoperation	17.2±9.2	17.4±5.9	0.787
Final follow up	18.9±9.4	17.6±6.0	0.674
p value (pre-final)	<0.001	<0.001	
PI-LL mismatch (°)			
Preoperation	21.6±20.4	23.4±22.6	0.412
Postoperation	4.3±9.7	2.0±10.3	0.306
Final follow up	5.1±10.8	3.7±11.6	0.262
p value (pre-final)	<0.001	<0.001	
SVA (mm)			
Preoperation	75.5±52.0	77.3±58.2	0.287
Postoperation	22.1±46.6	20.4±46.2	0.272
Final follow up	29.8±47.9	25.3±48.4	0.189
p value (pre-final)	<0.001	<0.001	
Lumbar Cobb angle (°)			
Preoperation	23.8±11.8	25± 9.6	0.529
Postoperation	9.8±8.9	9.4±7.7	0.552
Final follow up	10.2±9.0	9.6±7.8	0.424
p value (pre-final)	<0.001	<0.001	

### Clinical outcomes

Comparison of the clinical outcomes revealed no significant differences between the two groups (Table 3). The operative time (mean = 548.3 vs. 421.6 minutes) and estimated blood loss (mean = 1,851.1 vs. 1,552.4 ml) were higher in the ALIF than OLIF group. The preoperative VAS and ODI scores were similar for both groups (*P* = 0.662). The postoperative and final follow–up VAS scores were significantly improved in both groups (*P* < 0.001) (Table 3). There were no significant differences in VAS or ODI scores between the two groups.

**Table 3:** Comparisons of clinical outcomes between the two groups.

	ALIF	OLIF	P—value
VAS back score			
Preoperation	6.1± 2.7	6.2± 2.8	0.662
Postoperation	3.3± 2.3	3.3± 2.4	0.682
Final follow up	3.0± 2.5	3.1± 2.6	0.296
p value (pre-final)	<0.001	<0.001	
VAS leg score			
Preoperation	4.4± 3.5	3.5 ± 3.3	0.33
Postoperation	2.2± 2.4	1.2 ± 2.2	0.47
Final follow up	2.1 ± 2.6	2.2 ± 2.4	0.83
p value (pre-final)	<0.001	<0.001	
ODI			
Preoperation	44.4± 14.3	46.1± 15.5	0.74
Postoperation	49.5± 15.1	50.1± 18.3	0.4
Final follow up	31.2 ± 17.6	30.2 ± 16.2	0.478
p value (pre-final)	<0.001	<0.001	
SRS-22, pain score			
Preoperation	2.5 ± 0.6	2.3 ± 0.6	0.496
Postoperation	2.5 ± 0.6	2.5 ± 0.7	0.974
Final follow up	3.0 ± 0.8	3.2 ± 0.9	0.162
p value (pre-final)	<0.001	<0.001	
SRS-22, self-image score			
Preoperation	2.7 ± 0.6	2.8 ± 0.7	0.18
Postoperation	3.5 ± 0.5	3.5 ± 0.5	0.864
Final follow up	3.4 ± 0.8	3.6 ± 0.7	0.153
p value (pre-final)	<0.001	<0.001	
SRS-22, mental health score			
Preoperation	3.6 ± 0.7	3.6 ± 0.8	0.968
Postoperation	3.8 ± 0.6	3.6 ± 0.7	0.239
Final follow up	3.8 ± 0.8	3.8 ± 0.8	0.85
p value (pre-final)	<0.001	<0.001	
SRS-22, satisfaction score			
Preoperation	3.0 ± 0.9	2.9 ± 0.7	0.432
Postoperation	4.0 ± 0.6	4.0 ± 0.5	0.812
Final follow up	3.8± 0.9	4.1 ± 0.4	0.758
p value (pre-final)	<0.001	<0.001	
SRS-22, function score			
Preoperation	2.9 ± 0.6	2.7 ± 0.6	0.648
Postoperation	2.8 ± 0.4	3.2 ± 0.5	0.845
Final follow up	3.3 ± 0.7	3.5 ± 0.7	0.182
p value (pre-final)	<0.001	<0.001	
SRS-22, total score			
Preoperation	2.9 ± 0.5	2.7 ± 0.6	0.445
Postoperation	3.3 ± 0.4	3.4 ± 0.8	0.53
Final follow up	3.4 ± 0.5	3.6 ± 0.7	0.113
p value (pre-final)	<0.001	<0.001	

The incidence of complications was higher in the ALIF than OLIF group (45% vs. 31%) (Table 4). There were two vessel injuries in the ALIF group. There were two cases of transient thigh pain and/or numbness in the ALIF group and two of transient thigh flexion weakness in the OLIF group. Thigh pain and numbness diminished within 2 weeks of surgery. None of the patients had any persistent motor or sensory deficits after surgery. In the ALIF group, there were two cases of dural tear and four of postoperative infection; two cases of postoperative infection required operative intervention. One case of dural tear and three of postoperative infection occurred in the OLIF group; none of these patients required operative intervention. The numbers of implant failures were similar between the two groups. However, the case with rod fracture in the ALIF group required revision of the hardware.

**Table 4:** Summary of complications.

	ALIF	OLIF
Implant failure	5(13%)	3(8%)
Proximal vertebra fracture	2(5%)	1(3%)
Screw pullout	2(5%)	2(5%)
rod fracture	1(3%)	0(0%)
Pseudarthrosis	2(5%)	2(5%)
Perioperative complications	10(26%)	6(16%)
Infection	4(11%)	3(8%)
Neurological	2(5%)	2(5%)
Vascular	2(5%)	0(0%)
Dura tear	2(5%)	1(3%)

## Discussion

ASD is associated with sagittal and coronal plane malalignment. Positive sagittal and coronal imbalance is a strong predictor of pain and disability [1–3]. Restoration of sagittal and coronal imbalance is the primary goal of ASD surgery [1–3]. Deformity correction has traditionally involved a combined anterior/posterior approach or a posterior-only approach. Mild ASD can be treated with posterior release (facet osteotomy) and posterior fusion via a posterior approach [26]. However, facet osteotomy is often inadequate because stiff, collapsed disc spaces limit posterior shortening. Posterior approaches require exposure of the dura and nerve roots, placing them at greater risk of injury. In addition, placement of interbody cages via the posterior approach can be difficult when performing revision surgery in patients with significant scarring and bone grafting that may have altered the anatomy [27].

Moderate or severe ASD typically requires anterior disc space augmentation and/or posterior 3–column osteotomy [28]. Posterior three–column osteotomy provides excellent sagittal correction of severe ASD, with up to 35° LL restoration and 10cm posterior trunk translation. Even in experienced hands, however, posterior three–column osteotomy carries significant immediate (neurological deficits, durotomy) and delayed (infection, pseudarthrosis) surgical risks [7, 9, 11, 29]. In addition, ALIF is a good option for treating moderate or severe ASD. ALIF has several advantages, including enabling direct decompression of neural foramina, providing accessibility to L5–S1, requiring less mobilization of the psoas muscle (which lowers the risk of lumbar plexus injury), and enabling resection of the anterior longitudinal ligament, wide discectomies, and insertion of wedge–shaped lordotic grafts for massive correction of deformity. However, ALIF also poses risks, including bowel injury, ileus, vascular injury, hernia, ureter injury, lymphedema, lymphocele, and retrograde ejaculation [3, 10, 12, 24, 30].

In recent years, with advances in surgical techniques and instrumentation, minimally invasive surgeries have been introduced. In particular, minimally invasive LLIF has been increasingly used as an alternative to ALIF [14, 20]. Compared to ALIF, LLIF can avoid injury to the abdominal viscera and peritoneal penetration, and can reduce the risk of injury to the great vessels, including the common iliac vein, inferior vena cava, and iliolumbar vein, as well as the sympathetic chain [24, 26]. Compared to TLIF/PLIF, LLIF has lower risks of dural tear injury, nerve root injury, and paraspinal muscle injury [27, 28]. Furthermore, wide cages that support the lateral rims of the endplate can be placed via the lateral approach, which may prevent subsidence and subsequent loss of deformity correction [3, 13, 15, 24, 31–34].

Minimally invasive LLIF is classified into DLIF and OLIF. DLIF is performed via a lateral retroperitoneal transpsoas approach. It is associated with a risk of injury to the lumbar plexus and the psoas muscle [6, 35–40]. In contrast, OLIF is performed by an oblique retroperitoneal psoas–preserving approach. This has several advantages, including less invasion of the psoas muscle and neural structures, and good access to the lower lumbar levels [3, 21, 22, 24]. Therefore, we use OLIF as a treatment option for ASD.

In some of our cases, lumbar interbody fusion from L1 to S1 was performed. If access to lower lumbar levels is restricted by a high–riding pelvis, a separate incision and/or position change are required [20–24]. However, we performed lumbar interbody fusion from L1 to S1 without a separate incision or position change by tilting the operating table in the 45° right oblique decubitus position [24].

In this study, we assessed and compared the radiographic data, complications, and HRQoL scores of patients with ASD who underwent ALIF+PSF or OLIF+PSF.

The radiographic results showed good overall correction in both groups. In a retrospective review of 43 consecutive patients with adult scoliosis treated using a lateral approach, Sharma et al. reported that the mean correction was 3.7° at each accessed level in the coronal plane for a total of 87 instrumented levels [41]. Similarly, there was a mean gain of 2.8° of lordosis at each level. Anand et al. reported that a mean preoperative Cobb angle of 22° was corrected to 7° (68% reduction) [4]. Similarly, in the present study, the mean Cobb angle decreased from a preoperative value of 23.8° to 10.2° at the last follow–up (57% reduction) in the ALIF group, and from a preoperative value of 25.0° to 9.6° at the last follow–up (61.6% reduction) in the OLIF group.

Recent studies involving treatment of ASD have focused on sagittal spinopelvic alignment rather than coronal alignment [2, 3, 24, 27]. These studies showed that global sagittal alignment is the most critical factor, with patients having a positive sagittal balance showing significantly worse pain, function, and self–image. Pelvic parameters can also influence outcomes. SVA and PT are correlated with HRQoL outcomes [42, 43]. Increased PT is associated with worse pain and function. In addition to PT, the LL–PI relationship has been analyzed. LL–PI mismatch < 11° has been suggested to positively influence outcomes [44]. These studies suggest that the ideal radiographic parameters are SVA < 50 mm, PT < 20°, and LL–PI mismatch < 10°.

In this study, radiographic parameters were evaluated in both groups. The mean SVA, preoperatively and at the last follow–up, was +75.5 and +29.8 mm in the ALIF group, and +77.3 and +25.3 mm in the OLIF group, respectively. The LL, preoperatively and at the last follow–up, was 30.5° and 48.5° in the ALIF group and 28.3° and 48.7° in the OLIF group, respectively. There were good radiological outcomes (SVA < 50 mm, LL-PI mismatch < 10°) in both groups. All radiographic parameters were similar between the two groups.

Surgery for ASD is associated with a significant risk of complications [10, 26, 27]. In the present study, the OLIF group had fewer complications, which may reflect the less invasive nature of the technique used (Table 4).

Overall, both groups showed significant clinical improvement in sagittal radiographic deformity parameters and HRQoL. Both groups had significant decreases in VAS back and leg pain scores. There were no significant differences in the ODI or VAS scores between the two groups, suggesting similar improvement, as per the outcomes reported for traditional open ASD surgery [2, 3, 24].

This study had some limitations, the first of which was its retrospective nature. In addition, the choice of surgery was based mainly on surgeon preference; there were no specific criteria for selecting the surgical technique (ALIF or OLIF). These two techniques were used throughout the study period, but OLIF was introduced later than ALIF. To minimize the influence of surgical technique on outcomes, one senior surgeon performed all operations. However, this study included a large cohort from a single institution. Further multicenter prospective studies are required to confirm the results.

## Conclusion

OLIF+PSF achieved similar radiographic and HRQoL outcomes in ASD to ALIF+PSF. However, the rates of complications were higher for the ALIF than OLIF approach. Therefore, OLIF is a safe and reliable surgical treatment option for ASD.
